# Targeting bone in cancer therapy: Advances and challenges of bisphosphonate-based drug delivery systems

**DOI:** 10.5599/admet.2756

**Published:** 2025-06-08

**Authors:** Mohammadmahdi Eshaghi, Fariba Ganji, Hossein Shaki, Lobat Tayebi

**Affiliations:** 1 Biomedical Engineering Group, Faculty of Chemical Engineering, Tarbiat Modares University, P.O. Box: 14115-143, Tehran, Iran; 2 Institute for Engineering in Medicine, Health, & Human Performance (EnMed), Batten College of Engineering and Technology, Old Dominion University, Norfolk, VA, 23529, USA

**Keywords:** Nanomedicine, tumour, targeted drug delivery, nanomaterials

## Abstract

**Background and Purpose:**

Bisphosphonates (BPs) are well-known for their strong affinity toward bone mineral matrices and are widely used to inhibit excessive osteoclast activity associated with various bone disorders. Beyond their clinical use, their unique bone-targeting capability has positioned them as promising ligands for drug delivery systems aimed at treating bone-related cancers.

**Approach:**

The review analyses published studies on BP-functionalized drug delivery systems, including direct drug conjugates, calcium-based nanomaterials, carbon-based nanostructures, and self-assembling systems such as micelles and liposomes. In vitro assays (*e.g.* hydroxyapatite binding, cell viability) and in vivo biodistribution studies are discussed to evaluate targeting efficiency and therapeutic outcomes. The impact of BP structure, linker chemistry, and carrier material on drug release and bone accumulation is examined.

**Key Results:**

BP-functionalized systems consistently demonstrate improved bone targeting and enhanced drug accumulation at tumour sites compared to non-targeted approaches. Both direct conjugates and nanocarrier-based systems show promising results, with some formulations offering controlled drug release and reduced systemic toxicity. Despite these advances, certain challenges such as burst release and incomplete clinical validation remain.

**Conclusion:**

This review highlights the significant progress in BP-based drug delivery platforms for bone cancer therapy, demonstrating their potential to concentrate therapeutic agents at bone tumour sites while minimizing off-target effects. The integration of nanotechnology with BP targeting offers new opportunities for treating bone metastases and primary bone tumours. However, further research is needed to address current limitations and translate these findings into clinical practice.

## Introduction

**Figure 1.**
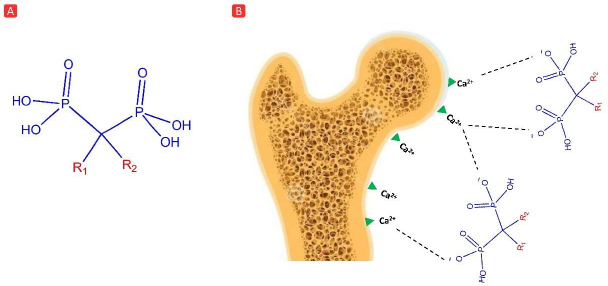
(A) General chemical structure of BPs; (B) demonstration of BPs affinity for calcium ions

Bisphosphonates (BPs) are a class of compounds with high affinity to bone mineral matrix and the ability to inhibit accelerated bone resorption [1,2]. The extracellular matrix of bone is composed of organic and inorganic sections. While the organic portion is rich in filamentous proteins and glycosaminoglycans, hydroxyapatite (HAp) crystals account for the majority of the inorganic portion [3]. These crystals, having large amounts of calcium ions in their structure, show a high binding affinity for BPs [4]. The structure of BPs is characterized by a P=C=P backbone. The central carbon atom is linked to two phosphonate groups and two other groups referred to as R_1_ and R_2_. Figure 1 exhibits the general structure and action of BPs.

The identity of the R_1_ and R_2_ groups differentiates between different BPs. Table 1 introduces R_1_ and R_2_ Groups for the most common BPs. These groups play a major role in the affinity of BPs to HAp [5]. For instance, the presence of an amine group as the R_2_ group of alendronate (ALN) results in the formation of a hydrogen bond with HAp crystals, which extends BP-HAp interaction [6]. Furthermore, the two OH arms of each phosphonate group in a BP molecule form an ionic crosslinked network with a Ca^2+^ ion in the structure of HAp, which is chiefly responsible for the affinity of BPs to the mineral network [7]. With the same logic, in cases where the R_1_ group is a hydroxyl, ionic interaction with Ca^2+^ ions, and consequently, BP affinity to HAp is further increased [8].

**Table 1.** Chemical structure of commonly used BPs

Name	R_1_ group	R_2_ group
Alendronate	OH	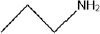
Zoledronate	OH	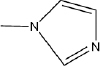
Pamidronate	OH	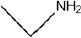
Risedronate	OH	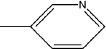
Clodronate	Cl	Cl
Ibandronate	OH	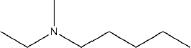
Etidronate	OH	CH_3_

Therefore, BPs can be used to target ligands and deliver drugs to the bone microenvironment through their binding with the HAp of bone tissue. Given these attributes, they have been recognized as valuable therapeutic agents for bone disorders characterized by abnormal activity of osteoclasts. A primary example of such disorders would be osteoporosis [9,10]. When the balance between osteoclast activity and osteoblast activity is shifted to the benefit of the former, bone resorption increases and bone tissue density decreases, which leads to the risk of pathological fractures [11]. Studies on BP pharmacodynamics have revealed how they ameliorate this condition. Initially, BPs selectively accumulate in the bone mineral network. The acidic medium of bone tissue with extra-normally active osteoclasts causes the removal of BPs from the mineral network. The BPs molecules are then endocytosed by osteoclast cells, which are metabolized into a toxic metabolite, which triggers cell apoptosis [12]. Currently, there are many bisphosphonate-based comercialized drugs in the market for different bone disorders (Table 2).

**Table 2.** List of commercialized BPs for bone disorders

Brand name	Active ingredient	Application	FDA approval year	Company
Didronel	Etidronate	Paget’s disease, Irregular Bone Growth	1987	MGI Pharma
Aredia	Pamidronate	Hypercalcemia of Malignancy, Paget’s disease, Bone damage caused by breast/bone marrow cancer	1991	Pfizer
Fosamax	Alendronate	Osteoporosis	1995	Merck
Skelid	Tiludronate	Paget’s disease	1997	Sanofi
Actonel	Risedronate	Osteoporosis, Paget’s disease	1998	Procter & Gamble Pharmaceuticals
Zometa	Zoledronate	Hypercalcemia of Malignancy, Multiple Myeloma	2002	Novartis
Boniva	Ibandronate	Osteoporosis	2003	Roche Therapeutics
Fosamax Plus D	Alendronate / Cholecalciferol	Osteoporosis	2005	Merck
Actonel with Calcium	Risedronate / Calcium Carbonate	Osteoporosis, Paget’s disease	2005	Procter & Gamble Pharmaceuticals
Aclasta	Zoledronate	Osteoporosis, Paget’s disease	2007	Novartis
Reclast	Zoledronate	Osteoporosis	2007	Novartis
Atelvia	Risedronate	Osteoporosis	2010	Sanofi
Binosto	Alendronate	Osteoporosis	2012	EffRx Pharmaceuticals SA

Aside from traditional bone disorders such as osteoporosis and Paget’s disease, which involve high bone turnover, tumour development in bone tissue is another condition that leads to extreme osteoclast activity. Studies have revealed that upon the formation of a tumour in bone tissue, osteoclasts and cancerous cells contribute to the growth and activity of each other. Cancerous cells release certain growth factors in bone tissue that promote osteoclast activity and osteoclast differentiation. Similarly, osteoclasts release growth factors that contribute to tumour cell growth, proliferation, and metastasis [13]. In addition to anti-osteoclast activity, BPs have been reported to exhibit anticancer properties. They interfere with the process of cell migration, adhesion, and angiogenesis. Moreover, they can have synergistic anticancer effects with chemotherapeutic agents and elicit immune responses against tumours [14,15].

Given their dual role as osteoclast inhibitors and well-established targeting ligands for the bone mineral matrix, along with their demonstrated anticancer effects, BPs present a promising avenue for designing drug delivery systems to treat various types of bone-related cancers. Not only can they be employed to deliver an anticancer agent to the site of bone tumours selectively, but also their pharmacodynamic properties can be leveraged to control osteoclast activity and prevent pathological fractures in the tumour-bearing bone. For these reasons, BPs have been a major area of focus for researchers in the past decade. This review provides a comprehensive analysis of the literature on drug delivery systems designed using BPs for targeted drug delivery to bone tumours. The literature reports have been categorized based on the class of the drug delivery systems. Furthermore, the review highlights the major advantages and limitations of bisphosphonate-based drug delivery systems and offers the authors' perspective on future directions for bone-targeting therapies.

## Bisphosphonate-drug conjugates

Due to its straightforward preparation process, the direct conjugation of drugs with a bisphosphonate molecule, utilizing cleavable bonds under specific physiological conditions, has become a widely adopted strategy in bone-targeted drug delivery. Table 3 summarizes the details of various bisphosphonate conjugated systems. In this table, the position of the BPs in the bisphosphonate-drug complex is reported as either an ‘outside network’ or a ‘within network’. The former indicates that the majority of bisphosphonate molecule arms, including OH groups, R_1_ and R_2_, are exposed and free for binding to the bone matrix, while the latter means that most of these arms are engaged with drug molecules to form the conjugate. The assumption is that the “outside networks” are more favourable for targeting purposes as they have more free arms for binding to the bone matrix. Nevertheless, multiple studies have reported that “within networks” have effective bone-targeting properties. Interestingly, systems that engage all OH arms of bisphosphonate, for forming the complex, have demonstrated satisfactory in vivo distribution [16-19].

**Table 3.** Structural details and targeting efficiency of BP-drug conjugate systems

Drug name	Bisphosphonate name	Type of conjugation	BPs position****	Outcome	Ref.
Doxorubicin (DOX)	Alendronate	Hydrazone bond	Outside	Highly efficient bone targeting *in vitro* and *in vivo*	[20]
5-Fluorodeoxyuridine (5F)	Alendronate	Non-cleavable covalent bond	Outside	Low therapeutic impact due to non-cleavable linker	[21]
Platinum isopropyl amine (PtC3)	Alendronate / Pamidronate	Chelation by P=O arms	Outside	PMD targeting function maintained after conjugation	[22]
Palladium II	Alendronate	Ionic conjugation	Within	More than 95 % binding to HAp *in vitro*	[19]
Pt(NO_3_)_2_(en)	BP-15*	Ionic conjugation	Within	Selective accumulation in hard tissues over soft tissues	[17]
Pt(NO_3_)_2_(en)	BP-15	Ionic conjugation	Within	Selective accumulation in hard tissues over soft tissues	[18]
DOX	12b80**	Hydrazone bond	Outside	Highly efficient bone targeting *in vitro* and *in vivo*	[23]
Bortezomib (BTZ)	BP-22***	Boronate ester bond	Outside	No direct bone affinity assay, biological tests support effective bone targeting	[24]

* (BP15): 2-amino-1-hydroxyethane-1,1-diyl-bisphosphonate

** (12b80): DOX-conjugated BP-21

*** (BP-22): 2-(bis(2-hydroxyethyl)amino)ethyl(bis(diethoxyphosphoryl)methyl)carbamate

**** (BPs Position): the position of the BPs in the bisphosphonate-drug complex has been reported as either ‘outside network’ or ‘within network’

Schem *et al.* [21] developed a non-cleavable conjugate between ALN and an antimetabolite drug, 5-fluorodeoxyuridine (5F), and assessed the compound’s application for treating breast cancer bone metastasis. *In vitro*, a cell viability assay was performed for multiple breast cancer cell lines, which were treated with free 5F, free ALN, or ALN-5F conjugate. Free 5F and free ALN exhibited stronger cytotoxicity than ALN-5F. Experiments by nude mice revealed that free ALN is superior to free 5F and ALN-5F in terms of reducing tumour size *in vivo*. Authors attributed the lack of superior therapeutic results for ALN-5F compared to free ALN and free 5F to the non-cleavable nature of the bond between ALN and 5F, which hinders the pharmacodynamic properties of both agents.

Ye *et al.* [20] conjugated hydrazine and monoethyl adipate (HYD), using it as a pH-sensitive linker to conjugate doxorubicin (DOX), as the anticancer drug and ALN, as the targeting ligand (DOX-HYD-ALN). The hydrazone bond is among the most widely applied linkers for pH-controlled drug release. According to the HAp binding assay, over 80% of DOX-HYD-ALN was bound to HAp in 90 minutes, which was 70% more than that of free DOX. In addition, *in vivo* biodistribution assay indicated a significant accumulation of DOX-HYD-ALN in tumour-bearing bone and negligible concentrations in other organs. These results demonstrate the effective targeting efficiency of ALN and promise fewer severe side effects. *In vivo* studies using nude mice revealed that DOX-HYD-ALN can decrease tumour growth rate significantly compared to free DOX. The improved performance of the conjugated system compared to the free drug can be attributed to ALN-mediated targeting, which increases drug concentration in the tumour site.

Alvarez-Valdez *et al.* [22] prepared complexes made of two different BPs, ALN or pamidronate (PMD), as targeting ligand, and platinum isopropyl amine (PtC3), as the drug, (BP-PtC3). P=O arms of ALN and PMD acted as chelating agents to form a complex with PtC3. The goal here was to determine the targeting capability of the two complexes for bone mineral matrix. To this end, a ^1^H NMR test was used to assess the binding affinity of ALN-PtC3 and PMD-PtC3 for HAp. The methodology included incubating a defined concentration of each complex with varying concentrations of HAp, testing the supernatant with ^1^H NMR spectroscopy after centrifugation, and monitoring the changes in the NMR spectrum. ^1^H NMR analysis indicated that the affinity of PMD for HAp was not compromised as a result of forming a complex with PtC3. However, ALN lost its binding affinity for HAp after forming a complex with PtC3. Therefore, PMD can be used as a targeting ligand to deliver PtC3 to bone tissue.

**Figure 2.**
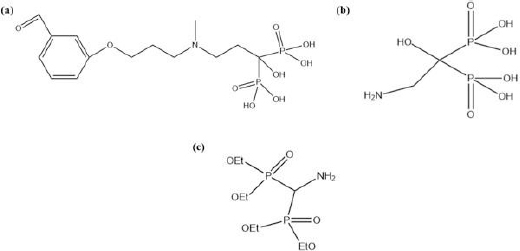
Chemical structure of (A) BP-21 (B) BP-15 (C) BP-22

David *et al.* [23] used a hydrazone-based linker to conjugate DOX and a synthetic bisphosphonate (BP-21) to obtain a pH-sensitive system, named 12b80. The chemical structure of BP-21 can be observed in Figure 2A. BP-21 was prepared from 3-(3-bromopropoxy)benzaldehyde in a 6-step procedure. As shown in Figure 2A, the novelty of BP-21 is in its R_2_ arm structure. Five out of six steps of the synthesis protocol are concerned with the preparation of this structure, which eventually turns 3-(3-bromopropoxy)benzaldehyde into 3-((3-(3-(1,3-dioxolan-2-yl)phenoxy) propyl)(methyl) amino) propanoic acid. In the final step, this compound reacted with tris(trimethylsilyl) phosphate in THF under an argon atmosphere to yield BP-21. An outstanding sustained release profile for DOX was offered by 12b80. Less than 15% of the drug was released after 3 days at pH 4. Under physiological pH, it was less than 2 %. *In vitro* binding of 12b80 to HAp and bovine bone was noticeably superior to free DOX. Similarly, much higher levels of 12b80 than free DOX accumulated in the tibia and tumour of *in vivo* animal models.

Nadar *et al.* [17] developed a radioactive complex of Pt(NO_3_)_2_(en), as a platinum-based drug and 2-amino-1-hydroxyethane-1,1-diyl-bisphosphonate (BP-15) (Figure 2B), as the targeting ligand (Pt-BP). BP-15 was obtained from a 3-step procedure, starting with the reflux of N-phthaloylglycine in SOCl_2_, followed by a reaction with tris(trimethylsilyl)phosphite in THF, ending with another reflux in an aqueous acidic solution. Pt-BP was formed via ionic interaction between the OH arms of BP-15 and the Pt^2+^ ion. *In vivo* biodistribution studies were conducted on mice to elucidate the targeting functionality of BP-15. Very low levels of Pt(NO_3_)_2_(en) accumulated in the tibia, femur, humerus, and spine, whereas Pt-BP accumulated significantly in these bones, which confirmed BP-15’s effective targeting functionality. Moreover, Pt-BP was excreted from soft tissues over 72 h of study, whereas the concentration of Pt(NO_3_)_2_(en) in soft tissues remained constant in this period. This result promises the reduction of side effects from the Pt-based drug as a result of forming a complex with BP-15.

Tao *et al.* [24] used three different pH-sensitive linkers to conjugate bortezomib (BTZ) and 2-(bis(2-hydroxyethyl)amino)ethyl(bis(diethoxyphosphoryl)methyl)carbamate (BP-22) for treating multiple myeloma (BP-BTZ). The chemical structure of BP-22 can be observed in Figure 2C. By modifying the chemical structure of the linkers, the release rate of BTZ was perfectly modulated.

A lead candidate with an optimum hydrolysis-mediated release rate was selected for further study. The selection was based on the superior *in vivo* antitumor activity compared with the two other candidates, which signifies the role of release rate in therapeutic efficiency. BP-22 increased the local concentration of BTZ in bone tissue and prevented quick clearance from bone marrow by the bloodstream. Additionally, this formulation offered the significant advantage of serum stability.

## Bisphosphonate-functionalized drug delivery systems

### Bisphosphonated calcium-based systems

Research on the biological activity of calcium-based materials such as HAp, calcium phosphate (CP), and bioactive glass (BG) has proven their promising application for bone regeneration [25-27]. Since the density of cancerous bone tissue is seriously compromised as a result of osteoclast cells’ stimulation by tumour cells, using such materials as a drug delivery system for anticancer drugs is a promising solution for the parallel treatment of bone tumours and bone regeneration.

Li *et al*. [28] used ibuprofen as a model drug for assessing ALN-conjugated HAp nanoparticles (NPs) as a drug delivery system. Compared to naked HAp NPs, ALN-modified NPs could encapsulate considerably higher amounts of the drug, which can be attributed to the ionic affinity between amine groups of ALN and carboxyl groups of ibuprofen (IBF). However, ibuprofen experienced a huge burst release (more than 90%) after just 3 hours of the *in vitro* experiment. This burst release can be associated with the fact that a hydrophobic drug was encapsulated in polar HAp NPs.

Chu *et al*. [29] modified CP NPs using an ALN-conjugated polyethylene glycol (PEG) moiety for delivering methotrexate (MTX), an anticancer drug, to tumour-bearing bone. Due to the acid-sensitive structure of CP NPs, about 90% of the payload was released under acidic conditions (pH 4.7) in the first 3 hours, followed by a steady pattern in the remaining 21 hours. Regarding bone targeting properties, the affinity of NPs for bone positively correlated with the amount of ALN used in the formulation.

In another study, Mehnath *et al*. [30] functionalized DOX-loaded BG NPs with hyaluronic acid (HA)-ALN conjugate. *In vitro* release experiments in acidic (pH 4.5) and neutral (pH = 7.4) environments revealed acid-sensitivity of the NPs. Under acidic conditions, NPs released no more than 65% of DOX and after 48 hours, no more drug was released. *In vitro* HAp binding assay revealed a boosted affinity of NPs to HAp after the inclusion of ALN in the formulation. HAp binding increased 4 times for ALN-modified NPs compared with naked NPs after 2.5 hours of incubation. *In vitro* cytotoxicity assay revealed time and dose-dependent toxicity of DOX-loaded BG NPs on the MG-63 cell line. DOX-loaded NPs were superior to free DOX in terms of cytotoxicity in all concentrations. Also, as the concentration increased, this superiority became more noticeable.

**Figure 3.**
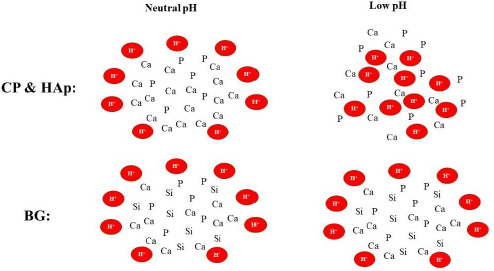
The lower sensitivity of BG to the acidic environment compared with CP and HAp

HAp and CP have similar composition, and both are labile to acidic conditions. According to the reviewed literature, it can be concluded that nanostructures based on these materials would readily collapse in acidic conditions and release their payload abruptly [28,29]. On the contrary, BG, which has a different composition than HAp and CP, was shown to be less labile to pH alterations and managed to maintain a more sustained release profile for its payload [30]. This outcome can be attributed to the difference between the chemical composition of BG, HAp, and CP as depicted in Figure 3.

A sustained release profile is crucial for enhancing the therapeutic efficiency of drug delivery systems in tumour therapy. Considering these factors, BG stands out as a more suitable candidate among the studied calcium-based materials for drug delivery applications where a sustained release profile is preferred.

Besides the acid-sensitive structure of Ca-based NPs, another reason for burst release in acidic conditions is that many studies have attempted to encapsulate hydrophobic drugs in such hydrophilic structures. Table 4 illustrates the correlation between the chemical structure of calcium-based drug delivery systems and their release profiles. As evident from the data, BG significantly outperforms CP and HAp in providing a more sustained release profile.

**Table 4.** Study of release profile of bisphosphonated Ca-based drug delivery systems

Carrier composition	Bisphosphonate name	Cumulative drug release profile	Ref.
HAp	Alendronate	>90 % in 3 h (pH not specified)	[28]
CP	Alendronate	>90 % in 3 h (pH 4.7)	[29]
BG	Alendronate	>65 % in 48 h (pH 4.5)	[30]
Calcium Ion	Risedronate	>100 % in 48 h (pH 5.5)	[31]
HAp	Medronate	>100 % in 48 h (pH not specified)	[32]
CP	Medronate	>100 % in 48 h (pH not specified)	[32]

### Bisphosphonated carbon-based systems

**Figure 4.**
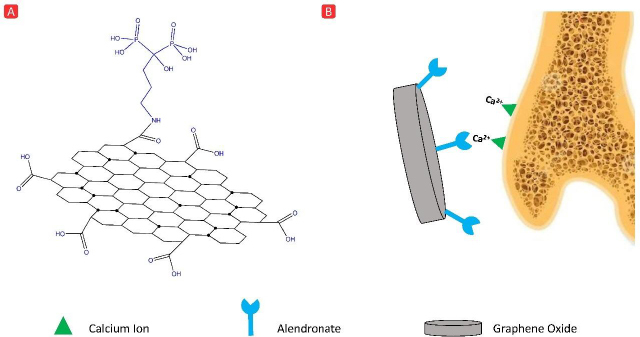
(A) Chemical structure of GON-ALN, (B) demonstration of GON-ALN affinity for calcium ions

Carbon-based systems refer to inorganic carbon-based structures such as graphene oxide and carbon quantum dots. Pham *et al*. [33] used ALN-functionalized graphene oxide nanosheets (GON) for DOX delivery to tumor-bearing bone (DOX@GON-ALN). The chemical structure and schematic illustration of this system are shown in Figure 4. Notably, GON-ALN yielded an impressive DOX loading capacity and encapsulation efficiency of 99.1 and 98.5 %, respectively. In a 3D collagen gel containing HAp and cancerous cells, ALN-functionalized nanosheets yielded noticeably lower cell viability (less than 50 %) compared to naked nanosheets, which signified the role of ALN for targeting purposes. *In vivo* imaging revealed preferential accumulation of DOX@GON-ALN in tumor-bearing bone rather than healthy bone, which can be associated with extreme HAp exposure in cancerous bone tissue. Such exposure makes the mineral matrix more available to BP ligands for binding [34,35].

Lee *et al*. [36] developed polymeric quantum dots (PQDs) by coating the surface of fullerene (C60) dots using HA. The presence of a variety of functional groups in the HA structure, coupled with a large surface area of quantum dots, facilitated its modification with multiple components, including ALN for bone targeting, RGD for integrin targeting, and Chlorin e6 (Ce6) for photodynamic therapy of bone tumour. The small size of quantum dots (smaller than 10 nm) is a major advantage and inhibits steric hindrance for the effective interaction of ALN and RGD with their receptors. *In vivo* biodistribution test using single-ligand formulations revealed that ALN is more effective than RGD in delivering PQDs to the site of tumour-bearing bone. Therefore, BPs should be prioritized over tumour-specific ligands like RGD for designing drug delivery systems for bone tumours. This research was one of the few with successful results in vivo and managed to reduce tumour volume in mice models.

### Bisphosphonated self-assembling systems

The formation of self-assembling systems relies upon the amphiphilic nature of the constituting molecules or macromolecules, which leads to the formation of a core-shell structure. Polymeric micelles are self-assembling systems that are being widely researched for drug delivery applications. In the aqueous medium of blood plasma, the hydrophobic end of the constituting molecules forms the core, and the hydrophilic end creates the shell [37,38]. Another class of self-assembling systems are liposomes, which are made of two layers of phospholipids. The hydrophobic tail and hydrophilic head of phospholipids form a hydrophilic core, a hydrophobic two-layer wall, and a hydrophilic shell. Therefore, liposomes can carry hydrophobic and hydrophilic drugs simultaneously. Several nanodrug products for cancer therapy based on liposomes have received FDA approval over the recent years [39,40].

Ye *et al*. [41] developed DOX, PEG, and ALN micelles for treating metastatic bone tumours. DOX and PEG were linked through a hydrazone bond to obtain a pH-sensitive system. As anticipated, ALN-modified micelle demonstrated higher binding affinity to HAp *in vitro* compared with naked micelle. According to the *in vivo* distribution test, the concentration of ALN-modified micelle in the liver and other major organs was much less than that of free DOX. The highest concentration of micelles was observed in bone tissue.

Zhu *et al*. [42] prepared self-assembling NPs for the treatment of bone metastasis using block copolymers consisting of PEG as the hydrophilic segment and Poly(L-lysine) (PLL) as the hydrophobic segment. While BTZ was grafted on the PLL chains using catechol as a pH-sensitive linker, ALN was conjugated to the PEG chains. An *in vitro* HAp binding assay was performed using different concentrations of ALN in the micelle. The binding positively correlated with the amount of ALN used in the formulation, verifying the BP's functionality. These results were further verified by *in vivo* experiments, as the concentration of ALN-modified micelle in the tumour site was considerably higher than that of naked micelle.

He *et al*. [43] prepared cisplatin-loaded coordination polymer NPs for treating bone metastasis of breast cancer. The formulation of the amphiphilic constituent included octadecyl succinic anhydride (OSA) as hydrophobic and PEG as hydrophilic segment (OSA-PEG). The NPs were functionalized with ALN (OSA-PEG-ALN). *In vitro* HAp binding assay confirmed the high affinity of OSA-PEG-ALN for HAp. *In vivo* experiments indicated that OSA-PEG-ALN accumulated in the tumour site rather than in other organs. Also, the accumulation level was much higher than that of naked NPs.

Feng *et al*. [44] encapsulated DOX in liposomes, which were functionalized with ALN-HA ligand via a disulfide linker. This linker would, in turn, lead the liposomes to bone mineral matrix and tumour cells, followed by intracellular redox-sensitive release of DOX. The body weight of mice models treated with the final liposome remained perfectly stable, demonstrating minimal systemic side effects resulting from ALN-mediated targeting. ALN-containing liposomes exhibited higher accumulation in tumour sites *in vivo* compared with naked liposomes.

Xi *et al*. [45] functionalized HA-stearic acid micelles with ALN for osteosarcoma therapy using curcumin (CUR). As expected, the amount of ALN-functionalized micelle bound to HAp was much higher than that of a naked micelle. Importantly, the prepared micelle had a very low critical micelle concentration (CMC), which assured increased *in vivo* stability. This achievement is crucial for micelles since high CMC values could cause the collapse of the micelle structure after intravenous injection. This low CMC value can be associated with the proper choice of the hydrophobic segment with a partition coefficient that is large enough.

Wu *et al*. [46] modified DOX-loaded liposomes with ALN and heparin for the treatment of osteosarcoma and bone metastasis of breast cancer. Heparin has been reported to increase the blood circulation time of liposomes in addition to possessing anti-metastasis properties. NPs were prepared with two surface densities of ALN. Interestingly, NPs with smaller surface density accumulated more effectively in tumours *in vivo*. This could show that an optimized concentration of BPs needs to be determined for the synthesis procedure to obtain maximized bone targeting efficiency.

In Table 5, we have compiled reports that conducted *in vitro* HAp binding assays to establish a correlation between nanoparticle size and the targeting efficiency of BPs. It was concluded that BP-functionalized NPs with hydrodynamic diameters above 100 nm can bind to HAp more effectively than those with sizes below this value. It can be interpreted that larger NPs allow more effective interaction between BP ligands and HAp crystals. Moreover, it seems that polymeric micelles can bind to HAp more efficiently than lipid-based NPs.

**Table 5.** Hydrodynamic diameter and HAp binding efficiency of self-assembling NPs (Bisphosphonate name Alendronate)

Carrier type	Carrier composition	Hydrodynamic diameter, nm	*In vitro* HAp binding efficiency, %	Ref.
Lipid-based NP	DOPA, DOPC, OSA, PEG	>65	>40 in 1 h	[43]
	DOPA, DOPC, OSA, Cholesterol, PEG	>61	>20 in 1 h	[47]
	DOPA, DOPC, OSA, cholesterol, PEG	>65	Less than 15 in 2 h	[48]
	SPC, cholesterol, DDAB, PEG-S	>106	Over 80 in 1 h	[46]
Polymeric micelle	PEG, PLL	>63	>60 in 1 h	[42]
	Pullulan, PEG, PTX	>70	Over 40 in 1 h	[49]
	PEG, DOX	>114	>70 in 15 min	[41]
	Acetic anhydride, curcumin	161	>55 in 1 h	[50]

### Bisphosphonated PLGA nanoparticles

Nguyen *et al*. [51] modified PLGA NPs with ALN for bone-targeted DOX delivery. Since PLGA NP is relatively hydrophobic, whereas ALN is hydrophilic, a lipid was conjugated to ALN to act as an intermediate and form a stable ALN corona on the PLGA NP surface. This nanocarrier yielded high loading efficiency (>80 %). *In vitro* HAp binding assay revealed that ALN-modified NPs yield maximum HAp binding when used at an optimum concentration. At a concentration of 100 μg mL^-1^, 80 % binding was achieved, whereas increasing the concentration to 1 μg mL^-1^ reduced the binding to 20 %. Therefore, an optimized concentration of NPs should be used to obtain maximum HAp binding.

Kozlu *et al*. [52] used ALN-modified PLGA nanoparticles for co-delivery of DOX and Celecoxib (CXB). DOX and CXB have been shown to possess synergistic anticancer properties. *In vitro* HAp binding assay confirmed that the bone affinity of ALN is not compromised as a result of conjugation with PLGA NPs. Also, increasing the surface density of ALN improved HAp binding to a certain extent, and beyond a certain concentration of ALN, the affinity figure plateaued. Similar to the work of Wu *et al*. [46], this report verifies that the density of BP corona on the nanocarrier surface should be optimized for maximal bone targeting and excessive surface modification can yield opposite results.

Yuan *et al*. [53] used zoledronate (ZOL)-functionalized PLGA NPs for co-delivery of Gemcitabine (GCB) and Epirubicin (EPB). While GCB is used as a first-line treatment, EPB was added due to its wider therapeutic window. According to *in vitro* experiments, ZOL-functionalized NPs had almost as much affinity for bone powder as pure ZOL. This affinity also increased with time. After 2 and 8 hours of incubation with HAp, the binding percentage was roughly 50 and 90 %, respectively.

**Figure 5.**
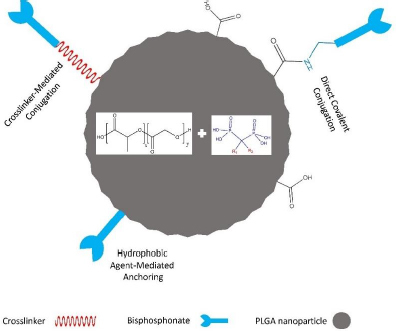
Strategies for Functionalizing PLGA NPs with BPs

These reports verify that modifying PLGA NPs with BPs does not affect the bone-targeting property of the latter. Since PLGA is FDA-approved for many biomedical applications, and considering maintaining the bone affinity of BPs despite covalent conjugation with PLGA, BP-modified PLGA NPs hold promise for developing drug delivery systems for bone tumours. Figure 5 demonstrates different approaches for modifying PLGA NPs with BPs.

## Key advantages and limitations of BP-functionalized drug delivery systems

Each class of drug delivery systems reviewed in the previous sections exhibited certain advantages and disadvantages. Most of these achievements are the consequence of structural and organizational relationships between BP molecules and other components of drug delivery systems. Table 6 summarizes distinguished achievements and unignorable drawbacks of the discussed literature reports. Considering all aspects of the reviewed literature, it could be concluded that BPs improve the bone-targeting efficiency and consequently therapeutic efficiency of all drug delivery systems for treating bone-related cancers, regardless of the type of carrier. However, focusing on some carriers such as PLGA and liposomes, which are already cleared by the FDA for biomedical applications, could accelerate the process and increase the chances of successfully passing clinical trials and receiving a final FDA-approved product.

**Table 6.** Distinguished achievements and drawbacks of the BPs-functionalized drug delivery systems

Drug delivery system type	Distinguished achievements	Unignorable drawbacks	Ref.
BP-Drug Conjugate (12b80/DOX)	Outstandingly sustained release profile, which improved therapeutic efficiency of the drug, successful modulation of linker degradation rate	Unsuccessful in decreasing tumour volume *in vivo*	[23]
BP-Drug Conjugate (BP15/ Pt(NO_3_)_2_(en))	Selective accumulation in bone with higher metabolic activity	Limited worldwide availability of raw materials	[17]
BP-Drug Conjugate (BP22/BTZ)	Successful modulation of linker degradation rate, Serum stability, Delayed clearance from well-perfused bone marrow	-	[24]
Ca-based System (HAp/ALN/IBF)	Very high encapsulation efficiency despite the opposite nature of drug and carrier (hydrophobic/hydrophilic), which is achieved because of ALN	Huge burst release due to opposite nature of drug and carrier	[28]
Ca-based System (BG/HA/ALN/DOX)	Controlled degradation rate by addition of HA-ALN ligand and consequent control over release pattern	Despite the acid-sensitivity of BG NPs, around 50% of the drug was released in physiological pH as well	[30]
Inorganic Carbon-based Systems (GON/ALN/DOX)	Outstandingly high loading and encapsulation efficiency, Selective accumulation in tumour-bearing bone, High release sensitivity to pH	Similar *in vitro* cytotoxicity of free DOX and DOX@GON-ALN	[33]
Inorganic Carbon-based Systems (C60/HA/RGD/ALN/Ce6)	Capable of including multiple ligands with various functions, Significant therapeutic results *in vivo* and tumour volume reduction, successful circumvention of biocompatibility concerns of CQDs	Complex preparation procedure due to the presence of multiple ligands	[36]
Dendrimer (PAMAM/HA/ALN/DTX)	Sequential targeting of bone mineral matrix and cancerous cell receptor by a single ligand using a pH-sensitive linker	Significant burst release is anticipated *in vivo* due to the parallel sensitivity of nanostructure to reducing agents and pH	[54]
Albumin NP (Albumin/ALN/DOX)	Size stability in fetal bovine serum over time, Selective accumulation in tumour-bearing bone and clearance from other organs, Tumour-targeting functionality of albumin	Unsuccessful in decreasing tumour volume *in vivo*	[55]
Self-assembling System (PEG/ALN/PTX)	Biological half-life improved 4-fold compared with free drug	Significant accumulation in the kidney due to renal excretion	[56]
Self-assembling System (DOPE/CHEMS/DSPE/ALN/DOX)	Modifying NPs with ALN reduced nonspecific accumulation in the spleen and liver and doubled accumulation in the tumour	Increased accumulation in liver and spleen compared with free DOX	[57]
Self-assembling System (SPC/Cholesterol/HA/ALN/DOX)	Sequential targeting of bone mineral matrix and cancerous cell receptor by a single ligand using a redox-sensitive linker, Minimal systemic side effects are anticipated	Unsuccessful in decreasing tumour volume *in vivo*	[44]
Self-assembling System (HA/C18/ALN/CUR)	Very low CMC value and consequently high *in vivo* stability	Unsuccessful in decreasing tumour volume *in vivo*	[45]
Self-assembling System (DOPA/ZnPc/ALN/BTZ)	Successful inhibition of tumour growth *in vivo*	Unsuccessful in decreasing tumour volume *in vivo*	[47]
Multimodal System (BaTiO_3_ / Fe_3_O_4_/RIS)	Engineered to execute various modes of cancer therapy	Complex synthesis protocol	[58]
Metal-organic Frameworks (ZIF-8/ZOL)	Simple synthesis protocol with mild conditions enabling delivery of sensitive biological molecules	High burst release associated with the pH-sensitive nature of the ZIF-8 structure	[59]
PLGA NP (PLGA/ALN/ZOL/EPB/GCB/DOX/CXB)	High loading efficiency, Preservation of drug molecules from intracellular degradation, Prevention of quick efflux, Inhibition of burst release by the nanocarrier even under acidic conditions, Promising therapeutic results *in vivo* and reduced tumour volume	-	[51-53]
Self-assembling System (PIP-BP ionizable lipids)	An extensive library of piperazine-based bisphosphonate ionizable lipids were prepared which enabled targeted delivery to the bone microenvironment *in vivo* following systemic administration	The ring structure formed through cyclization may result in the random orientation of the bisphosphonate group on the surface of nanoparticles either outward or inward	[60]
BP-Drug Conjugate (Cy5.5-ALN)	*In vitro* and *in vivo* studies demonstrated that Cy5.5-ALN targets mineralized components of bone-forming tumours	No report of therapeutic efficiency evaluation with a small molecule drug	[61]
BP-Drug Conjugate ()	The reactivity of the complexes with 5'-GMP, B- and G-quadruplex DNA models, and their cytotoxicity against a panel of human tumour cell lines was explored	No *in vivo* experiments were reported	[62]

PAMAM: Polyamidoamine; DTX: Docetaxel; PTX: Paclitaxel; DOPE: Cholesteryl hemisuccinate; CHEMS: Dioleoyl phosphatidylethanolamine; DSPE: 1,2-distearoyl-sn-glycero-3- phosphoethanolamine; SPC: Soybean phosphatidylcholine; C18: Octadecanoic Acid; DOPA: Dioleoyl phosphatidic acid; RIS: Risedronate; ZIF-8: A metal-organic framework composed of Zn^2+^ ions and imidazolate ligands

## Conclusion

BPs were first introduced as anti-osteoporotic agents approximately 40 years ago. Over the past decade, they have been successfully employed as bone-targeting molecules in the design of drug delivery systems. This review summarizes recent advancements in utilizing BPs to develop drug delivery systems for treating bone-associated cancers. Regardless of various types of preparation methods, including conjugates of BPs to different drug molecules, Ca or carbon-based systems, self-assembling micelles or liposomes, metal-organic frameworks, and PLGA nanoparticles, the evidence demonstrates that BP-based drug delivery systems hold significant promise, particularly for the effective treatment of osteosarcoma. Despite the known side effects of BPs, their integration with conventional therapies offers enhanced targeting and cytotoxicity against bone cancer cells, presenting a high potential for synergistic effects that could improve therapeutic outcomes for many treatments.
